# MICRO-CREDENTIALS IN GERONTOLOGY EDUCATION: ENHANCING EXPERTISE OR THREATENING EXISTENCE?

**DOI:** 10.1093/geroni/igae098.1001

**Published:** 2024-12-31

**Authors:** M Aaron Guest, Suk-hee Kim

**Affiliations:** Arizona State University, Phoenix, Arizona, United States; Northern Kentucky University, Highland Heights, Kentucky, United States

## Abstract

As a field, gerontology faces the challenge of preparing a workforce to address the complex needs of an aging population. Yet, in the twenty-first century, gerontology has faced many challenges in accomplishing this goal. Academic programs continue to face closure, combination, and student recruitment challenges. This is despite national and global aging. Ensuring continued access to gerontology-related content has become central for many educators. A movement from independent programs and classes to gerontology content infused throughout the curriculum has been one approach. Another has been the development of micro-credentials. These programs offer the opportunity to demonstrate competency in an area through a short, focused program of study. They are characterized by their specificity, flexibility, and accessibility and may offer a viable solution for rapidly equipping professionals with gerontological competencies. They differ from formal degree programs and continuing education, although they can be components of each. In addition, they cost fractions of a traditional degree program, often have no requirements for admission, and usually can be completed through self-paced learning. Yet, learners have limited guidance in deciding what constitutes a viable micro-credential. Indeed, micro-credentials are offered through a continuum provider, including academic-based institutions, nonprofit organizations, for-profit providers, and even employers. As such, it is unknown what many of these programs provide education, what skills they provide their learners, and if the content is scientifically accurate. While these programs can undoubtedly assist in infusing gerontological competency throughout the workforce, they also pose a severe threat to ensuring a trained and competent workforce.

